# Maxillary reconstruction with subperiosteal implants in a cancer patient: A one-year follow-up 

**DOI:** 10.4317/jced.59331

**Published:** 2022-03-01

**Authors:** Pablo Garrido-Martínez, Norberto Quispe-López, Néstor Montesdeoca-García, Germán Esparza-Gómez, José-Luis Cebrián-Carretero

**Affiliations:** 1 DDS, MsC, phD. Associate Professor, Department of Prosthesis, Faculty of Dentistry, University Alfonso X el Sabio, Madrid. Department of Oral and Maxillofacial Surgery, Hospital La Luz, Madrid; 2 DDS, MsC, phD. Associate Professor, Department of Surgery. Faculty of Medicine, Dental clinic. University of Salamanca; 3 DMD, phD. Chief, Department of Oral and Maxillofacial Surgery, Hospital La Luz, Madrid; 4 MD, DDS, phD. Professor Titular, Faculty of Odontology, University Complutense of Madrid, Madrid; 5 DMD, DDS, phD. Chief, Department of Oral and Maxillofacial Surgery, Hospital La Luz, Madrid Chief, Department of Oral and Maxillofacial Surgery, Hospital Universitario La Paz, Madrid

## Abstract

**Introduction:**

To describe a clinical case on cancer patient with ablative tumor surgery, from treatment planning, surgical resection and subsequent implantological rehabilitation.

**Case Report:**

A 61-year-old male, diagnosed with a squamous cell carcinoma in the maxilla, requires the removal of the lesion and corresponding oral rehabilitation. However, two surgeries were necessary to rehabilitate the upper jaw. A custom-made prosthesis was fabricated. It was made from sintered titanium using machined subperiosteal implants with a universal external connection. Finally, a milled cobalt- chrome structure was produced and a feldspar ceramic covering was subsequently applied.

**Conclusions:**

Rehabilitation using subperiosteal implants may be an alternative tool for complex surgery involving large atrophies or cancer patients who have undergone highly ablative surgery.

** Key words:**Oral rehabilitation, oral cancer, subperiostal implants.

## Introduction

The use of endosseous dental implants to replace missing teeth has been a highly predicTable solution over the years, and is now one of the primary techniques for dental rehabilitation (1). However, sufficient bone quantity and quality is needed for their placement. In cases of severe bone resorption, more advanced surgery is required for bone regeneration, which may involve higher rates of complications, morbidity and longer treatment times (2).

Subperiosteal implants were developed in Sweden at the beginning of the 1940s. Subperiosteal implants were custom-made fixtures that were inserted below the periosteum and stabilised with screws and the mucous tissue covering them (3). They were made of cobalt-chrome or titanium alloys and were connected to the prosthesis using transmucosal abutments that emerged into the oral cavity (4). While they were used for years to treat cases of maxillary atrophy, they were replaced with the endosseous implants designed by Brånemark (5). This was due to the complexity of the production process for subperiosteal implants. An impression of the residual alveolar ridge had to be taken and sent to the laboratory, where the structure was designed. The result was often an imperfect fit, due to the relative instability of this type of implant. Positioning these implants in the patient was very difficult and could cause a range of complications (6).

Further, advances in the field of oral and maxillofacial surgery have enabled cancer patients’ health to be restored. (7) In 1989, Hidalgo was the first to use a microvascular fibula flap for mandibular reconstruction after tumour resection (8). This continues to be one of the main techniques used for bone reconstruction today, due to its great versatility.

However, anatomy is significantly altered after cancer surgery. Therefore, the introduction of osseointegrated implants has been a genuine revolution in these treatments, given that they enable effective implant-supported or implant-retained dental rehabilitation, thereby recovering patients’ oral functionality (9).

The field of diagnosis and planning for these diseases has now greatly improved, enabling us to achieve greater benefits from treatments for our patients.

The aim of this article is to describe a clinical case concerning a cancer patient, from initial diagnosis to treatment plan, including surgical resection and dental rehabilitation, after overcoming several complications during the process.

## Case Report

**Figure 1:**
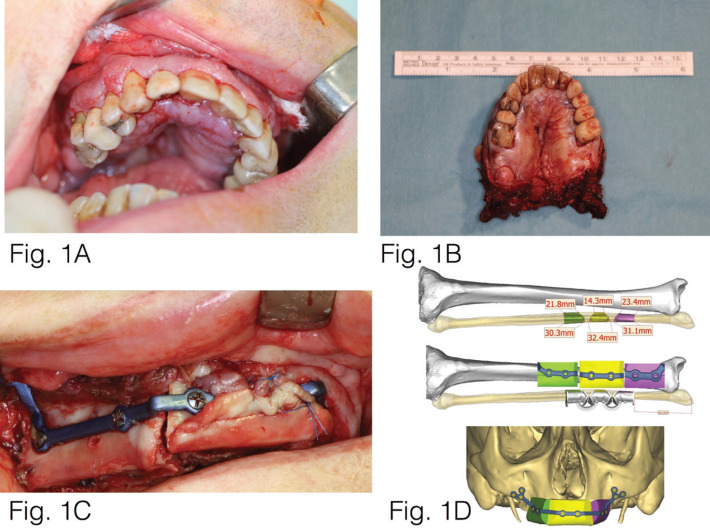
A: Intraoral photograph showing lesion in upper jaw. B: Resection piece after excision of the lesion. C: Microvascularized fibula flap to be transferred to the oral area under reconstruction, showing the position of the fibular artery. D: Virtual planning for the placement of a microvascular fibula flap with three osteotomies.

**Figure 2:**
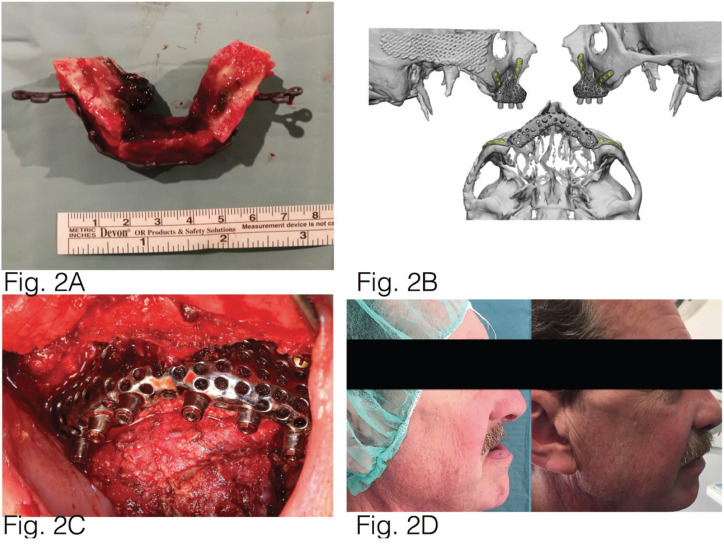
Fibula graft failure occurred due to thrombosis of the vascular anastomosis. B: Virtual planning for placement of a customised prosthesis. C: The customised prosthesis was positioned, which was fixed into the nasomaxillary and zygomaticomaxillary buttresses. D: Images show the before and after of the surgery.

We present the clinical case of a 61-year-old male, diagnosed with a squamous cell carcinoma in the maxilla (Fig. 1). Virtual planning was performed using digital software for the placement of a microvascular fibula flap with three osteotomies and a preformed plate (Fig. 2).

**Figure 3:**
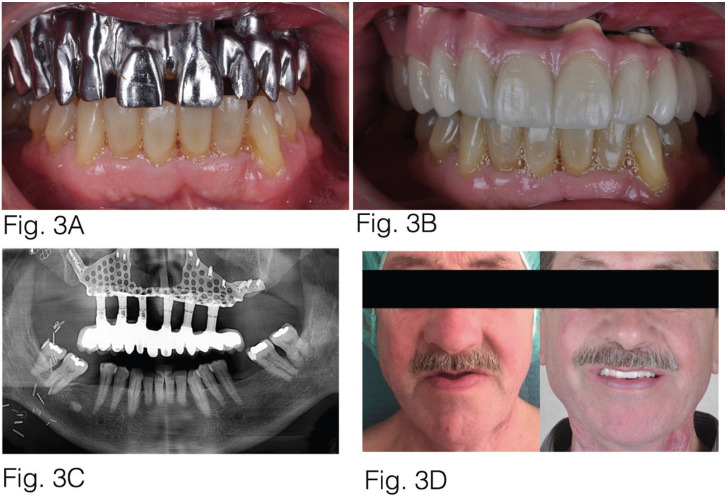
Intraoral photograph shows the milled cobalt-chrome structure. B: Intraoral photograph shows the final prosthesis. C: Ortopantomography showing the subperiostal implants and the definitive prosthesis. D: Images show the before and after of the definitive prosthesis.

Under general anaesthesia, complete surgical resection of the maxilla was performed, given that the tumour was multicentric (Fig. 3). The jaw was reconstructed using a microvascular fibula flap in three fragments. Virtual planning was used both for the resection and reconstruction cutting guides and production of the custom-made titanium plate to fix the fibula in the middle third of the face.

Seventy-two hours after the surgery, fibula graft failure occurred due to thrombosis of the vascular anastomosis and could not be recovered through revision of the anastomosis.

The flap was removed and reconstruction was performed using a titanium mesh moulded to the premaxillary anatomy to maintain the space, and the temporalis flap on the right. This patient did not need adjuvant administration of radiotherapy and chemotherapy.

Nine months later, virtual planning (Materialise Mimics v22.0®) was again performed for placement of a customised prosthesis. Designed by Avinent®, it was made from sintered titanium using machined subperiosteal implants with a universal external connection (4.1mm wide). To place the prosthesis, using general anaesthesia and nasotracheal intubation, an approach was made over the existing temporalis flap in the palate and dissection of maxillary buttresses was performed. The existing mesh was removed and the customised prosthesis was positioned, which was fixed into the nasomaxillary and zygomaticomaxillary buttresses with the help of a transcutaneous guide and additional screws in the median line area. Without subsequent complications, the patient was discharged and referred to the hospital’s Dental Service for metal-ceramic implant-supported dental restoration.

Using local anaesthetic, the subperiostal implants were found to be covered in mucous, and straight and 30º angled transepithelial abutments (MultiUnit®) were placed to create an optimal emergency prosthetic and secure the connections at the juxta-gingival level. After the tissue around the implants had healed, a splinted open tray impression was taken. Passive fit of the implants was tested and 3D printed test dentures were created to achieve optimum aesthetic and occlusal parameters. A milled cobalt-chrome structure was produced and a feldspar ceramic covering was subsequently applied.

## Discussion

Implant rehabilitation in patients with severe maxillary atrophy has always posed a challenge to the surgeon. Advances in diagnosis and planning, and improvements in regeneration techniques and material design have improved outcomes for these extremely complex cases. Nevertheless, complications may arise in this type of surgery, increasing morbidity, and the length and cost of the treatment for the patient (10).

For oral cancer patients to whom it has been necessary to administer radiotherapy, the placement and survival of implants may pose a risk (11).

Subperiosteal implants were used very frequently in the mid-1950s, until endosseous implants appeared, which are easier to place and rehabilitate (12). However, the digital revolution in the field of medicine and dentistry has been a great step forward in planning and processing customised structures for implant rehabilitation (13).

In 2009, Imburgia published a clinical case study on a subperiosteal implant rehabilitation, using CAD/CAM technology to produce a stereolithographic model in epoxy resin, which was subsequently sent to the laboratory to cast the structures (14). In recent years, laser sintering – an additive technique for manufacturing a range of titanium and cobalt-chrome structures – has been the method used for processing these subperiosteal implants.

In 2016, Cohen published an *in vitro* study on the biological behaviour of subperiosteal implant structures made from Ti6Al4V, produced by means of laser sintering and post-machining on various surfaces (15). They displayed a high level of bone-to-implant contact, with vertical growth demonstrated through histology and histomorphometry.

Cerea and Dolcini’s (16) retrospective clinical study is that which includes the largest number of patients rehabilitated using this technique. The study was conducted on 70 patients with two years of follow-up. These patients underwent partial or complete maxillary and mandibular rehabilitation with subperiosteal implants manufactured on laser sintered structures and subsequently polished through electroerosion, resulting in totally smooth surfaces. The implant survival rate was 95.8% and the main postoperative complications were pain, discomfort and swelling. There was an 8.9% rate of prosthetic complications.

In 2020, Mangano and colleagues (17) published a study of 10 patients, focusing on the posterior sectors of atrophic mandibles. These were rehabilitated by means of subperiosteal implants manufactured on laser sintered structures and subsequent decontamination and sterilisation with organic acids. After one year, none of the implants had been lost and all complications were minor.

In our case, after a year of follow-up there have been no operational or prosthetic complications. Quarterly check-ups have been performed on the patient, with periodontal and prosthetic assessments and, after one year, the structure was removed in order to fully clean the oral mucosa.

Rehabilitation using subperiosteal implants has improved significantly in recent years, due to the great advances created by digital planning and CAD/CAM. While further studies are needed, this may be an alternative tool for complex surgery involving large atrophies or cancer patients who have undergone highly ablative surgery.

## References

[1] Srinivasan M, Meyer S, Mombelli A, Müller F (2017). Dental implants in the elderly population: a systematic review and meta-analysis. Clin Oral Implants Res.

[2] Lim G, Lin GH, Monje A, Chan HL, Wang HL (2018). Wound Healing Complications Following Guided Bone Regeneration for Ridge Augmentation: A Systematic Review and Meta-Analysis. Int J Oral Maxillofac Implants.

[3] Weiss CM, Reynolds T (2000). A collective conference on the utilization of subperiosteal implants in implant dentistry. J Oral Implantol.

[4] Linkow LI (2000). Use of a tripodal mandibular subperiosteal implant with ramus hinges for facial asymmetry. J Oral Implantol.

[5] van Steenberghe D, Branemark PI, Quirynen M, De Mars G, I Naert I (1991). The rehabilitation of oral defects by osseointegrated implants. Journal of Clinical Periodontology.

[6] Moore DJ, Hansen PA (2004). A descriptive 18-year retrospec- tive review of subperiosteal implants for patients with severely atrophied edentulous mandibles. The Journal of Prosthetic Dentistry.

[7] Schou S, Pallesen L, Hjørting-Hansen E, Pedersen CS, Fibæk B (2000). A 41-year history of a mandibular subperiosteal implant. Clinical Oral Implants Research.

[8] Hidalgo DA (1989). Fibula free flap: a new me- thod of mandibule reconstruction. Plast Reconst Surg.

[9] Kokosis G, Schmitz R, Powers DB, Erdmann D (2016). Mandibular Reconstruction Using the Free Vascularized Fibula Graft: An Overview of Different Modifications. Arch Plast Surg.

[10] Barone A, Ricci M, Mangano F, Covani U (2011). Morbidity associated with iliac crest harvesting in the treatment of max- illary and mandibular atrophies: a 10-year analysis. Journal of Oral and Maxillofacial Surgery.

[11] Schiegnitz E, Al-Nawas B, Kämmerer PW, Grötz KA (2014). Oral rehabilitation with dental implants in irradiated patients: a meta-analysis on implant survival. Clin Oral Investig.

[12] Esposito M, Ardebili Y, Worthington HV (2014). Interventions for replacing missing teeth: different types of dental implants. Cochrane Database Systematic Reviews.

[13] Joda T, Zarone F, Ferrari M (2017). The complete digital workflow in fixed prosthodontics: a systematic review. BMC Oral Health.

[14] Imburgia M, Logozzo S, Hauschild U, Veronesi G, Mangano C, Mangano FG (2017). Accuracy of four intraoral scanners in oral implantology: a comparative in vitro study. BMC Oral Health.

[15] Cohen DJ, Cheng A, Kahn A, Aviram M, Whitehead AJ, Hyzy SL (2016). Novel osteogenic Ti- 6Al-4V device for restoration of dental function in patients with large bone deficiencies: design, development and implementa- tion. Scientific Reports.

[16] Cerea M, Dolcini GA (2018). Custom-Made Direct Metal Laser Sintering Titanium Subperiosteal Implants: A Retrospective Clinical Study on 70 Patients. Biomed Res Int.

[17] Mangano C, Bianchi A, Mangano FG, Dana J, Colombo M, Solop I (2020). Custom-made 3D printed subperiosteal titanium implants for the prosthetic restoration of the atrophic posterior mandible of elderly patients: a case series. 3D Print Med.

